# Scale up design study on process vessel dimensions for ultrasonic processing of water and liquid aluminium

**DOI:** 10.1016/j.ultsonch.2021.105647

**Published:** 2021-06-23

**Authors:** Mohammad Khavari, Abhinav Priyadarshi, Tungky Subroto, Christopher Beckwith, Koulis Pericleous, Dmitry G. Eskin, Iakovos Tzanakis

**Affiliations:** a Faculty of Technology, Design and Environment, Oxford Brookes University, Oxford OX33 1HX, UK; b Department of Materials, University of Oxford, Parks Rd, Oxford OX1 3PH, UK; c Brunel Centre for Advanced Solidification Technology, Brunel University London, Uxbridge UB8 3PH, UK; d Computational Science and Engineering Group, University of Greenwich, 30 Park Row, London SE10 9LS, UK; e Tomsk State University, 36 Lenin Avenue, Tomsk 634050, Russia

**Keywords:** Ultrasonic processing, Cavitation, Aluminium casting, Resonance length, Spectral analysis

## Abstract

Scaling up ultrasonic cavitation melt treatment (UST) requires effective flow management with minimised energy requirements. To this end, container dimensions leading to the resonance play a crucial role in amplifying pressure amplitude for cavitation. To quantify the importance of resonance length during the treatment of liquid aluminium, we used calibrated high-temperature cavitometers (in the range of 8–400 kHz), to measure and record the acoustic pressure profiles inside the cavitation-induced environment of liquid Al and deionized water (used as an analogue to Al) excited at 19.5 kHz. To achieve a comprehensive map of the acoustic pressure field, measurements were conducted at three different cavitometer positions relative to the vibrating sonotrode probe and for a number of resonant and non-resonant container lengths based on the speed of sound in the treated medium. The results showed that the resonance length affected the pressure magnitude in liquid Al in all cavitometer positions, while water showed no sensitivity to resonance length. An important practical application of UST in aluminium processing concerns grain refinement. For this reason, grain size analysis of UST-treated Al-Cu-Zr-Ti alloy was used as an indicator of the melt treatment efficiency. The result showed that the treatment in a resonance tank of ${L=\lambda }_{Al}$ (the wavelength of sound in Al) gave the best structure refinement as compared to other tested lengths. The data given here contribute to the optimisation of the ultrasonic process in continuous casting, by providing an optimum value for the critical compartment (e.g. in a launder of direct-chill casting) dimension.

## 1 Introduction

Ultrasonic cavitation melt treatment (UST) is an environmentally friendly, economical and sustainable technique [1], [2], [3] that has been shown to benefit the microstructure and mechanical properties of metallic alloys upon casting. The salient mechanism is related to the phenomenon of acoustic cavitation that aids structural refinement by heterogeneous nucleation through the activation of the solid particles and substrates [1], [2], deagglomeration [4], dispersion [2], [5], [6], fragmentation [7], and also leads to degassing of the treated melt [8]. The acoustic pressure amplitude and distribution generated by the immersed sonotrode and the resultant extent of cavitation region are the main process parameters that need to be controlled and optimised.

The beneficial effects of UST on the microstructure refinement of metals and its potential in manufacturing composite materials by dispersing inclusions have triggered a lot of interest among the metal manufacturing, aerospace and automotive industries, particularly due to the ease of implementation, versatility and effectiveness of the process. Optimisation requires deep understanding of the parameters affecting the process. However, inherent difficulties such as the opacity of the liquid metals and (until recently) lack of high temperature experimental tools that enable direct measurement of cavitation activity, limited the understanding of acoustic bubble dynamics and associated flow field effects. This imposes difficulties in process optimisation and control and eventually scaling up for adoption by industry. Typically, the effectiveness of UST is assessed through indirect post-process observations, such as microstructure analysis (e.g. grain size analysis [2], size and distribution of primary particles [9], [10]) and degassing efficiency [11].

So far, controlling UST in industrial processes is rather qualitative and intuitive; current efforts are concentrated on 1) characterizing the cavitation regime and the acoustic pressure spectrum in a range of frequencies and 2) achieving high efficiency in processing large melt volumes by using a single ultrasonic source coupled with a cleverly designed flow arrangement. The latter is a challenging task that requires, for example, strategically fitted partitions to exploit resonance leading to higher pressures, excite a larger liquid volume and increase the melt treatment residence time within the cavitation zone [12], [13], [14]. This approach showed promise in direct-chill casting experiments [15], [16]. Nevertheless, for a technological step up, we feel a more systematic study of the salient physical phenomena is required. There have been recent studies in optimising an ultrasonic reactor [17], [18], albeit these have been mostly done for low-temperature liquids.

A powerful tool in analysing and optimising the cavitation dynamics via synchrotron observation [19], [20], [21] (where only small-scale experiments are feasible and observations restricted in small volumes) is by measuring acoustic pressure emissions within the sonicated volume. Only handful of studies has been conducted so far on the characterization of cavitation-induced acoustic emissions in molten metals using high-temperature advanced sensors [2], [3], [14], [22], [23], [24], [25], [26], [27]. The recent advent of direct measurements of the acoustic pressure and cavitation intensity using cavitometers - calibrated for a broad range of frequencies - has opened new avenues for characterizing UST in various metallic melts, enabling insights in the fundamental governing mechanisms and the optimisation of melt processing.

Previous acoustic pressure measurements in liquid Al have mostly been confined to narrow-band frequency domains i.e. 15–50 kHz (with excitation frequency in the range of 17–24 kHz) [3]. These cover the effect of the driving frequency and up to the second harmonic and the corresponding ultra-harmonic emissions, partially neglecting the sub-harmonics and higher order harmonics and prominent peaks plus broadband components (associated with cavitation bubble collapses and non-linear/transient cavitation) that are important for the characterization of different cavitation regimes and precise quantification of generated acoustic pressures.

It has been reported [25] that the cavitation-induced pressure intensity in liquid Al is much more pronounced than that in water (otherwise a reasonable analogue to liquid Al in cavitation behaviour). The effectiveness of UST also relies on the volume of the liquid metal being treated and is governed by the size of the tank utilized for processing. Lebon et al. [3] numerically studied and experimentally validated the effect of the experimental vessel size (around 1λ) on the pressure field in water and showed that the resonance may play role in the magnitude of acoustic pressure. This work was, however, limited to small vessels (with a length at 65 mm (~0.85λ) and 75 mm (1λ)) with water and narrow-band calibrated cavitometers (neglected the effect from cavitating bubbles). The dependence of the acoustic pressure on the resonance may be particularly important for the ultrasonic processing of flowing liquid aluminium (e.g. in a launder of direct-chill casting) when the processing (residence) time needs to be increased by all means. The acoustic pressure variation and dependence on the dimensions of the experimental tank or partition has, however, not yet been confirmed explicitly for processing of liquid Al.

To this end, the present work aimed at closing the existing knowledge gap, related to acoustic characterization of cavitation activity in liquid Al in vessels with resonant and non-resonant dimensions, using cavitometer pressure sensors calibrated for a wide frequency range of 8–400 kHz as in [24]. Deionized water (having the closest cavitation properties to liquid aluminium) was used for comparison. In order to map the full pressure domain, cavitometer measurements were taken at different positions in the process tank. The measured cavitation activity was delineated with respect to the observed frequency spectrum and conclusions have been made in relation to the choice of the treatment domain that would result in pressure field amplification, increased cavitation activity and presumed increased UST efficiency. As an additional measure in verifying the treatment efficiency in liquid Al, microstructural observations (i.e. average grain size) were performed for the samples cast after UST of the melt in tanks of different lengths (with fixed width and depth).

## 2 Methods and materials

### 2.1 Experimental setup

**Fig. 1:**
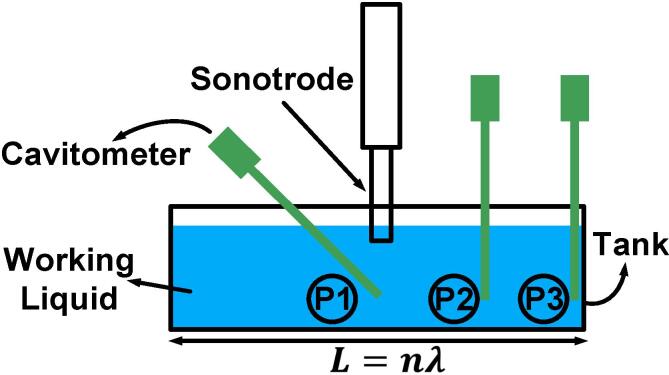
Experimental setup for pressure measurement via cavitometers. The sonotrode is immersed in the tank filled with working liquid (either DIW or liquid Al). The cavitometer is placed at three different positions, under the sonotrode (P1), quarter-length or middle (P2) and edge of the tank (P3).

Fig. 1 shows a schematic of the experimental setup. Two different working liquids (deionized water (DIW) and liquid Al) and various tanks for each liquid with dimensions L×H75×W100 mm (DIW) and L×H95×W100 mm (Al, based on industrial launders [28]) to maintain the same resonance and off-resonance conditions were deployed. Despite the different heights of the tanks, we made sure that liquid height in the vessels remained the same. The resonance length was defined as $L=n\lambda =c/f_{0}$ where $f_{0}$ is the driving frequency and c is the sound speed in the liquid (see Table 1). It is known that cavitation may change the speed of sound in the liquid in the vicinity of the sonotrode [29], though it was neglected in this study as there are currently no means to know what is the extent of this effect.

**Table 1:** Descriptions of experimental conditions.

Working Liquid	Tank Material	Vessel Length L/λ	Cavitometer Calibrated Range (kHz)	Driving Frequency*f*_0_ (kHz)	Sound Velocity c (m/s) [3]
DIW	Glass	0.7, 1, 1.3, 2	8–400	19.5	1482
Liquid Al	Refractory Board	0.5, 1, 1.1, 1.7, 2	8–400	19.5	4600

The following tank lengths were used: for liquid Al – L= 115, 230, 250, 390 and 460 mm corresponding to L=0.5λ, λ, 1.1λ, 1.7λ (for microstructure analysis) and 2λ; for DIW – L= 50, 75, 98 and 150 mm corresponding to L=0.7λ, λ, 1.3λ and 2λ, as $\lambda_{Al}=230$ mm and $\lambda_{DIW}=75$ mm. We had, therefore, selected two resonance lengths (λ and 2λ) and a number of off-resonance lengths based on the geometrical features of each container. In water, we could not consider a L=0.5λ measurement as the cavitometer device would not fit. Note that in order to have consistency in the measurements, we decided to use similar sonotrode diameter/resonance length ratio (based on the available commercial sonotrodes) for DIW and liquid Al (0.29 for DIW and 0.21 for Al). Table 1 summarises the experimental conditions. Sonication was applied using a 19.5 kHz 1-kW piezoelectric transducer (Hielscher Ultrasonics GmbH). The tank was filled with the working liquid and a ceramic (SiAlON) sonotrode (48 mm in diameter) for liquid Al and a Ti sonotrode (22 mm in diameter) for DIW were submerged in the centre of the tank, 10 mm below the liquid surface. The input power was adjusted to give a peak-to-peak displacement amplitude of 33 μm for both cases. The liquid level within the tank was maintained at 60 mm. A calibrated cavitometer [24] was immersed at three different positions inside the liquid, i.e., referring to Fig. 1, under the sonotrode (P1), at a quarter-length (P2) and at the edge of the tank (P3). Each experiment was repeated at least twice. For liquid Al experiments (except for the microstructure analysis), we used commercially pure 99.7 wt% Al, and a K-type thermocouple to constantly monitor the temperature during the experiments with all measurements recorded in the range of 710±30°C. The temperature was kept constant at 25 °C for DIW experiments.

**Fig. 2:**
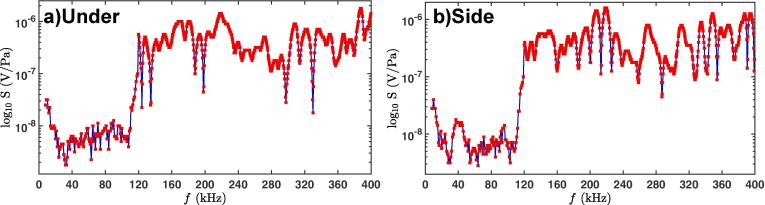
Sensitivity of the cavitometer vs. frequency (NPL) for two probe positions: a) under (position P1 in Fig. 1) and b) side (positions P2 and P3 in Fig. 1).

A digital oscilloscope PicoScope-3204D (Pico Technology) monitored and captured in real-time the acoustic pressure emissions as voltage signals that were amplified by a calibrated pre-amplifier. For each experiment, we recorded the acoustic data for 60 random waveforms of 2 ms each (with sampling rate of $500\times 10^{6}$ samples/s) under steady-state conditions to ensure the repeatability of the experiments and to account for the randomness of the cavitation phenomenon. The high temperature cavitometer with a spatial resolution of 40–50 mm and a bandwidth of up to 10 MHz [24] was calibrated over the range of 8–400 kHz at 0.5 kHz increments at the National Physical Laboratory (NPL), UK. The rationale behind this calibration range was to capture the associated emissions from collapsing bubbles at higher order frequencies as has been previously explained in the Introduction. Fig. 2 shows the calibration of the cavitometer as function of its sensitivity for the calibrated range (8–400 kHz) for two cases, under the sonotrode and to the side of it. The red markers are the calibrated data points for each discrete step and the blue lines are the interpolated values used for the intermediate frequencies. All pressure measurements were taken with respect to the ambient pressure, since the cavitometer had been calibrated at atmospheric pressure.

### 2.2 Microstructure experiments

As an indicator of ultrasonic melt treatment efficiency, the average grain size was measured from solidified samples cast from the melt subjected to UST. Grain refinement in Zr and Ti-containing aluminium alloys is known to be an indicator of UST efficiency [1], [2]. In this work, an Al-Cu-Zr-Ti alloy was prepared using a commercially pure aluminium alloy (99.7 wt% Al) with the addition of the alloying elements (i.e. Cu, Zr and Ti) through master alloys. The chemical composition of the alloy was determined by optical emission spectroscopy shown in Table 2. UST was performed using the same parameters and equipment as described above for liquid Al as we do not expect any noticeable variation in acoustic parameters of the alloy versus pure aluminium. Four experiments were carried out in tanks that were used for acoustic pressure measurements (as described in Section 2.1). One reference experiment was performed without UST, and three remaining ones were with UST at three different container lengths (L=λ, 1.7λ (off-resonance condition) and 2λ). For each experiment, the alloy was heated to 750°C and poured into the preheated tank, then UST was performed using the preheated sonotrode immersed approximately 10 mm into the melt. Experiments were repeated three times with consistent results. The treatment was performed for about 1 min, with liquid Al cooling down from 740±10°C to approximately 700°C then the melt was scooped using a preheated ladle and poured into a preheated steel mould. Microstructural samples were cut from the solidified cast, then mechanically ground, polished, and subsequently anodized using Barker’s solution (5% HBF4 in water solution) for approximately 60 s at 20 VDC. The grain size analysis was performed using the linear intercept method and statistical analysis was carried out afterwards.

**Table 2:** Average composition of the alloy obtained through OES.

Composition	Al	Cu	Zr	Ti
Amount (wt %)	Balance	4.1	0.16	0.06

### 2.3 From voltage to pressure: the deconvolution process

**Fig. 3:**
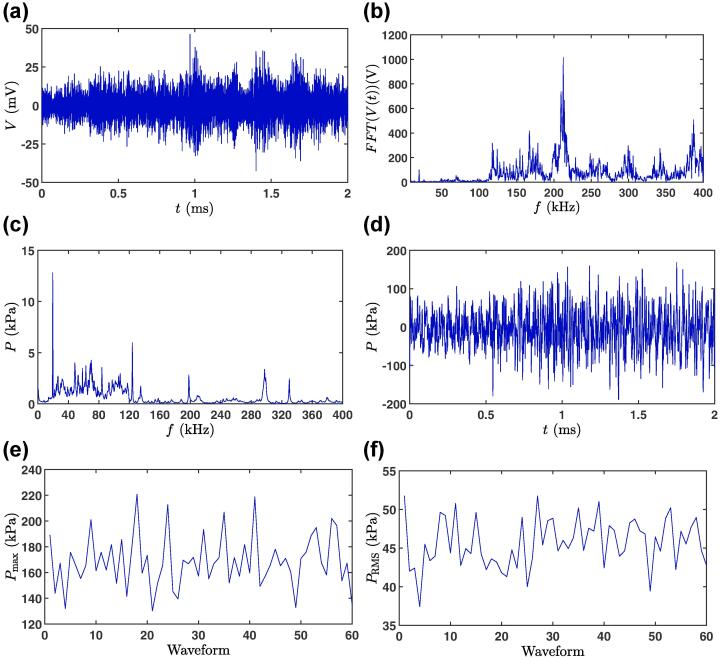
The deconvolution process: a) Original voltage signal. b) Fast Fourier Transform of (a). c) Pressure vs. frequency resulting from applying the sensitivity function of the cavitometer to the signal. The pressure was averaged over 60 waveforms. d) Pressure vs. time for one sample waveform. e) $P_{max}$ for each waveform from which the mean of maximum pressure is calculated. f) $P_{RMS}$ for each waveform from which the mean RMS pressure is found. Results obtained for DIW with the cavitometer at position P1 (under). Tank length was 75 mm (L=λ).

**Fig. A1:**
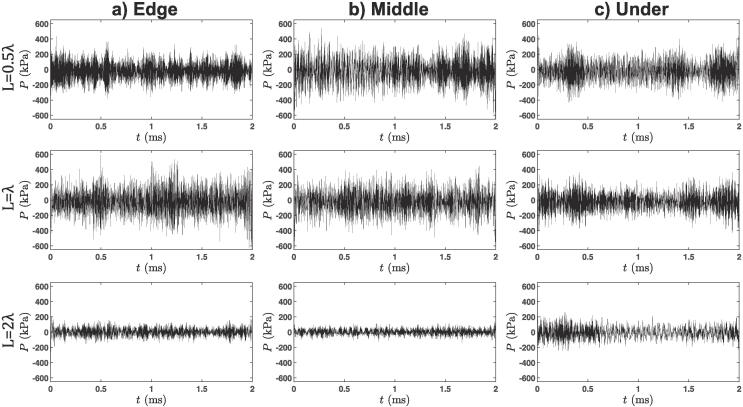
Pressure distribution in the time domain for a single random waveform for liquid Al for different cavitometer positions; edge (a), quarter length (middle) (b) and under (c), each for three tank lengthsL=0.5λ, λ and 2λ.

The entire analysis of the experimental acoustic data was carried out via an in-house MATLAB code based on the deconvolution process as described elsewhere [3], [30], [31], [32] and in Appendix D in Ref. [33] taking into account the cavitometer calibration. Fig. 3 shows the steps in the pressure conversion procedure for DIW as the working liquid, glass tank, cavitometer position P1 (under the sonotrode) and L=λ (tank length 75 mm). The noise (500μV, measured before the main experiments) was subtracted from the original voltage signals in the frequency domain recorded by PicoScope and low-pass filters were applied to avoid the contribution of the non-calibrated range of the cavitometer (outside the range of 8–400 kHz). We then used the Fast Fourier Transform (FFT) algorithm to compute the discrete Fourier transform of the input signal (Fig. 3(b)). The signal was then converted into a single-sided spectrum [33] to correct for the cavitometer sensitivity. After dividing the resultant signal by its corresponding sensitivity value at each frequency (Fig. 2), the signal was converted back to a double-sided spectrum, resulting in the pressure profile within the frequency domain (Fig. 3(c)). For each experiment, 60 random waveforms were recorded. The final frequency response of each experiment was the mean over 60 waveforms of the corresponding values for each waveform of 2 ms (Fig. 3(c)). An inverse Fast Fourier Transform would result in the pressure distribution in time domain [30], [31], [33]. Fig. 3(d) shows this final output of the deconvolution process as P-t profile for a single random waveform. The maximum ($P_{max}$) and root-mean-square (RMS) pressure ($P_{RMS}$) of each waveform were then calculated from these profiles. Fig. 3(e) and 3(f) show these values for 60 waveforms for the case study. The final $P_{max}$ and $P_{RMS}$ for each experiment were averaged over 60 waveforms. The pressure distribution in the time domain for a single random waveform for various probe positions and tank lengths for liquid Al is shown in Fig. A1 in the Appendix.

## 3 Results and discussion

### 3.1 Acoustic spectral analysis

**Fig. 4:**
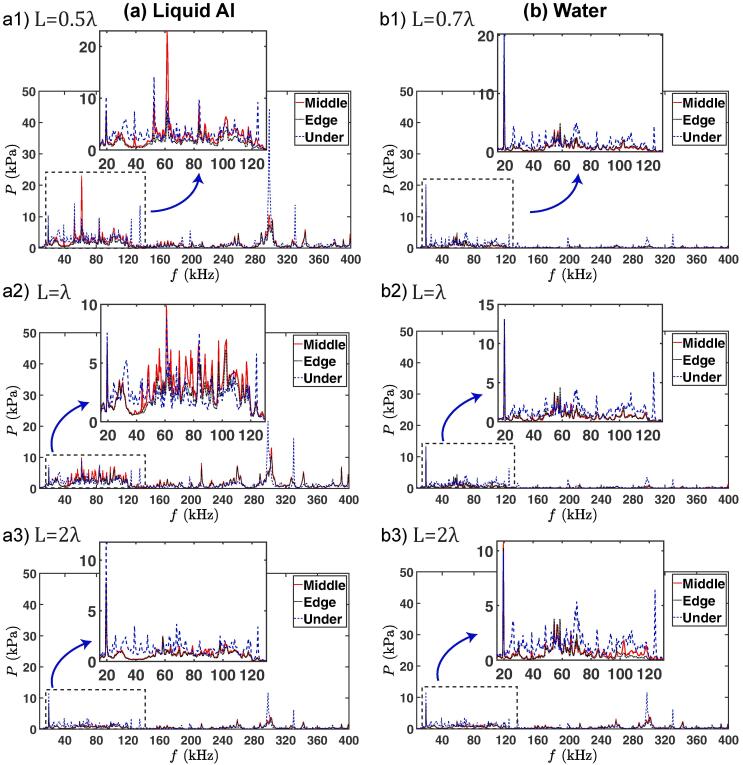
Pressure vs. frequency for three different tank lengths (L) for both liquid Al (a) and DIW (b), each for three different cavitometer positions; under, quarter-length and edge (colour online). Note the driving frequency and ultra-harmonics being captured by the cavitometer. Red solid line is for the pressure at quarter-length, dotted black line is for edge and dashed blue line is for under positions. The pressure for each case was averaged over 60 waveforms. Note different Y-axis scale in the insets. (For interpretation of the references to colour in this figure legend, the reader is referred to the web version of this article.)

**Fig. 5:**
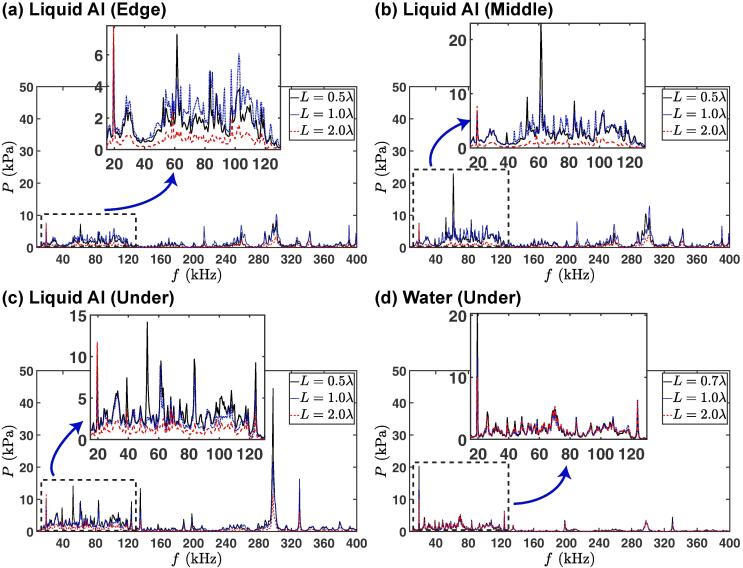
Pressure vs. frequency for three λ for different positions for liquid Al (a, b and c) and for positon ‘under’ for DIW (d) (colour online). Dotted blue line is for L=λ and dashed red line is for L=2λ for both liquid Al and DIW. Solid black line is for L=0.5λ for liquid Al and L=0.7λ for DIW. The pressure for each case was averaged over 60 waveforms. Note different Y-axis scale in the insets. (For interpretation of the references to colour in this figure legend, the reader is referred to the web version of this article.)

**Fig. 6:**
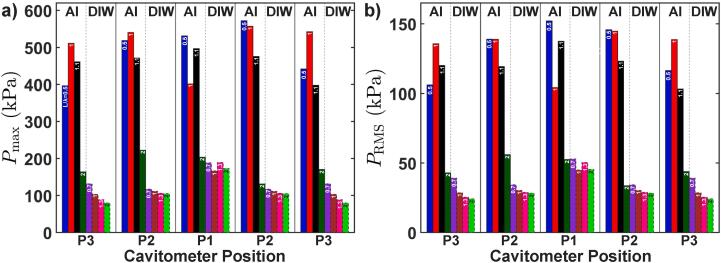
Bar plot of $P_{max}$ (a) and $P_{RMS}$ (b) for both liquid Al and DIW vs. the cavitometer position (see Fig. 1) for all resonace lengths for each liquid. Positions are under the sonotrode (P1), quarter-length or middle (P2) and edge of the tank (P3). The tank length for each case is shown as the L/λ ratio inside each bar. The four left bars for each sensor position belong to liquid Al and the four right bars belong to DIW. Note that the relative positions reflect different lengths of the tanks for each case.

**Fig. A2:**
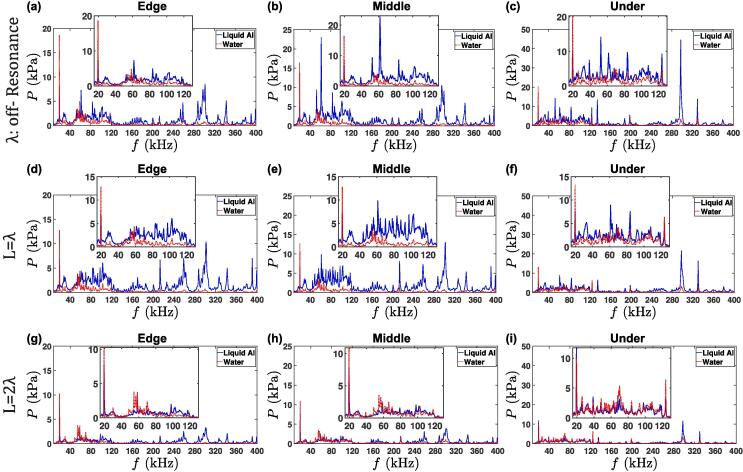
Comparison of pressure in frequency domain for DIW and liquid Al for different cavitometer positions and different tank lengths: off resonance (L=0.5λ for liquid Al and L=0.7λ for DIW, first row), L=λ (second row) and L=2λ (third row). The pressure for each case was averaged over 60 waveforms.

Fig. 4 shows the pressure in the frequency domain for three different values of L=0.5λ,λand2λ for liquid Al (a) and for L=0.7λ,λand2λ for DIW (b), each for three cavitometer positions: under, quarter length (middle) and edge. In retrospect, Fig. 5 compares the frequency response at various cavitometer positions for each L for liquid Al (a, b and c) and the position ‘under’ for DIW (d). Direct comparison of the frequency response of liquid Al and DIW is given in Fig. A2 in the Appendix. It was decided for the frequency spectrum comparison (Fig. 4, Fig. 5) to use only one off-resonance length (L<λ) for each case as it exhibits a higher pressure magnitude for both liquids (and among all the off-resonance lengths) in the majority of the studied cases (see Fig. 6).

The analysis of the frequency response for liquid Al in Fig. 4 shows that large pressure magnitudes were recorded under the sonotrode for every tank length. Specifically large pressure spikes were observed at high frequencies (300 kHz). For L=0.5λ (Fig. 4(a1)), the maximum peak pressure was ~ 45 kPa at 300 kHz under the sonotrode (P1), while the amplitude of the peak at this frequency reduced to 11 kPa for quarter-length (P2) (with the maximum peak pressure for this position calculated to be 23 kPa at 62 kHz), and to 8.5 kPa for edge (P3). At the driving frequency ($f_{0}=$19.5 kHz), the peak pressure for this L slightly dropped as the probe moved away from the sonication source and is 10.3, 6.8 and 7.3 kPa for P1, P2 and P3, respectively. For L=λ (Fig. 4(a2)), the maximum peak pressure for all three positions was at 300 kHz and was calculated to be ~ 22, 13 and 10 kPa for P1, P2 and P3 positions, respectively. The pressure at the driving frequency ($f_{0}=$19.5 kHz) for this L varied only slightly with position, being 7.5, 6.8 and 6.8 kPa for P1, P2 and P3, respectively. However, as we show in Fig. 4(a3), the peak pressures for L=2λ were significantly smaller than those in the shorter tanks. For L=2λ, the largest peak pressure for all probe positions was recorded at the driving frequency ($f_{0}=$19.5 kHz) as 11.7, 7.8 and 7.8 kPa for P1, P2 and P3, respectively. At the high frequency f=300 kHz for which we observed large spikes at smaller vessel lengths, the peak pressures are recorded to be 11.3, 3.9 and 3.3 kPa for P1, P2 and P3, respectively.

Unlike liquid Al, for DIW (Fig. 4b), no significant differences were observed for the pressure magnitudes or peaks for quarter-length and edge positions, while under the sonotrode, we observed higher pressure values and larger pressure spikes in the entire spectrum, a similar behaviour to liquid Al. Also, the maximum peak pressures for DIW were typically recorded at the driving frequencies of 19.5 kHz for all positions and lengths.

For DIW, the pressure magnitudes under the sonotrode seemed to be independent of L (Fig. 5(d)) and similarly there was no significant difference for the response for the other two positions (quarter-length and edge). This is in contrast with what was reported before [3]. The reason for this disagreement could be attributed to the narrow frequency range (15–50 kHz) of calibration as well as only short (L=λ or less) tanks used in the earlier study with the size of the sonotrode (48 mm) occupy most of the available space promoting strong shielding and attenuation of the acoustic emissions. Because for this range, the shielding and damping of the acoustic field was significant and the bubble emissions were not captured properly, while in the present study, a finer resolution in the spectral calibration range of 8–400 kHz allowed us to capture cavitation phenomena and contributions in more detail. The insensitivity to resonance length (vessel dimensions) in the case of water could be impactful for applications such as in 2D nanomaterials [34], or emulsions [35] where cavitation treatment is carried out in water. An observation that may potentially simplify the complexity of treatment vessel designs.

For liquid Al, as seen in Fig. 5(a)-(c), we observed a distinct drop in the pressure magnitudes for L=2λ in all positions, along with several resonance peaks at high frequencies (200–400 kHz) and a rise of the fundamental frequency component. However, these peaks were not significant for this large tank (2λ), considering that the rate of dissipation of energy is high in liquid Al [36]. Additionally, the fact of lower pressure peaks may indicate a smaller number of cavitating bubbles (bubble volume fraction) in the bulk liquid, allowing the sound wave at the driving frequency to reach farther distances (less shielding). This could be related to the absence of standing waves and corresponding antinodes that could promote bubbles growth/collapse (formation of bubbly clouds), for larger tanks (L=2λ) with liquid Al, which led to a pressure drop. It is worth noting that the peaks at higher frequencies for liquid Al have not been reported before, as previous studies did not use probes with this range of calibration.

For DIW, in contrast, the pressure peaks at high frequencies are suppressed due to the smaller lifetime of bubbles (compared to those in liquid Al) and, therefore, the driving frequency gives the most prominent peak (see Fig. 4(b) and Fig. 5(d)) with contributions from the cavitating bubbles. This could be the determining factor in defining the extent of the cavitation zone for liquid Al and DIW and can be complementary to the previous explanations that the borders of the cavitation zone were typically controlled by the acoustic impedance and the attenuation factor [24], [25].

On the other hand, for smaller tanks (L=0.5λ and λ) in liquid Al, the prominent peaks at higher frequencies overwhelmed the fundamental frequency. This is especially pronounced in the very active area of 50–100 kHz, represented by harmonics of the driving frequency and the corresponding ultra-harmonics as well as superimposed cavitation emissions from vigorous bubble oscillations and shock wave emissions from the inertial (transient) cavitation [37]. This observation is interesting as it clearly shows that in liquid aluminium for L<λ, and in contrast to water, the fundamental frequency does not give the prominent peak, with the rise of the 3rd harmonic (see Fig. 5 (a-c)) indicating a resonance mode that may be related to the harmonic cascading as explained in [38] or the non-linearity parameter of the host medium that promotes waveform distortions (similar to harmonic imaging technique where the resonance frequency from the body fluids is typically a multiple of the original frequency). In addition, the bubbles in smaller vessels (L<2λ) being confined to a smaller melt volume (compared to L=2λ), were expected to amplify the cavitation activity within this limited zone. This suggests a much higher bubble volume fraction for L<2λ than L=2λ. On the other hand, these populated bubbly structures could lead to a stronger shielding effect [27], [32], [39] and might as well interfere with the standing waves expected due to the resonance length and may suppress the number of antinodes [40] that would be considered beneficial for formation and collapse of bubbles. So the outcome is a trade-off between the two effects. Additionally, a more compact space of small tanks provided more reflections for the acoustic waves that could not be attenuated (as they could for larger tanks of L=2λ).

Furthermore, previous studies [19], [41] have shown that in liquid Al, cavitation bubbles behave in a non-linear stable manner and are capable of surviving in the molten liquid for long periods of time. This will amplify the intensity of the recorded cavitation signals at the bubble resonant frequency and improve the potential of cavitation treatment. This could be a reason for the observation of the prominent peaks in 200–400 kHz range for liquid Al (with 19.5 kHz excitation frequency), as seen in Fig. 4(a) and 5(a)-(c). The most plausible explanation is the oscillation of cavitation bubbles at their resonance size (with a natural frequency within this range of 200–400 kHz as shown below) or the periodic collapses (bubbles collapse and rebound for a few hundred of milliseconds as seen in [41]). The size of these oscillating cavitation bubbles in liquid Al can be estimated by the Minnaert equation [42], [43]: $f=1/2\pi \sqrt{\left[3\gamma P+2\left(3\gamma -1\right)\sigma /R_{0}\right]/\left(\rho R_{0}^{2}\right)}$ where γ is the gas specific heat ratio, ρ and σ are the density and surface tension of the liquid, P is the absolute pressure and f is the natural frequency of the oscillating bubbles. Solving this complete form of the Minnaert equation numerically for $R_{0}$ and using γ= 1.33, σ= 0.079 N/m and ρ= 1000 $kg/m^{3}$ [44] for water and γ= 1.41 [44], σ= 0.86 N/m and ρ= 2375 $kg/m^{3}$ [36] for liquid Al, the resonance radii of oscillating bubbles for all prominent frequencies (Fig. 4, Fig. 5) and their corresponding absolute pressures ($P=P_{\infty }+P_{RMS}$) from the time domain for the entire set of experimental data were estimated for both water and liquid Al and all three positions of the cavitometer.

The tabulated results are presented in Tables A1 (liquid Al) and A2 (water) in the Appendix. These estimates (excluding the driving frequency) give a range of 10–50 μm for the bubble radius for liquid Al, which is close to the previously reported theoretical linear resonance size of 20 μm at similar frequency of 20 kHz [3]. We do not observe any significant effect of cavitometer position on bubble radius. These calculated bubbles radii qualitatively represent the pressure peaks in the frequency domain captured by cavitometer during in-situ monitoring (as it is unlikely that the fundamental frequency in a relatively viscous and controlled by energy diffusion environment contributes to any peaks in the range of the 10^th^ to 20^th^ harmonic, i.e., 200–400 kHz). The bubbles in the cavitation zone are very unstable and multiply continuously, while most of the bubbles that are typically located outside the cavitation zone possess longer lifetimes, and their oscillations contribute to the emissions and rise of spectrum peaks at high frequencies.

### 3.2 Acoustic pressure measurements

**Fig. 7:**
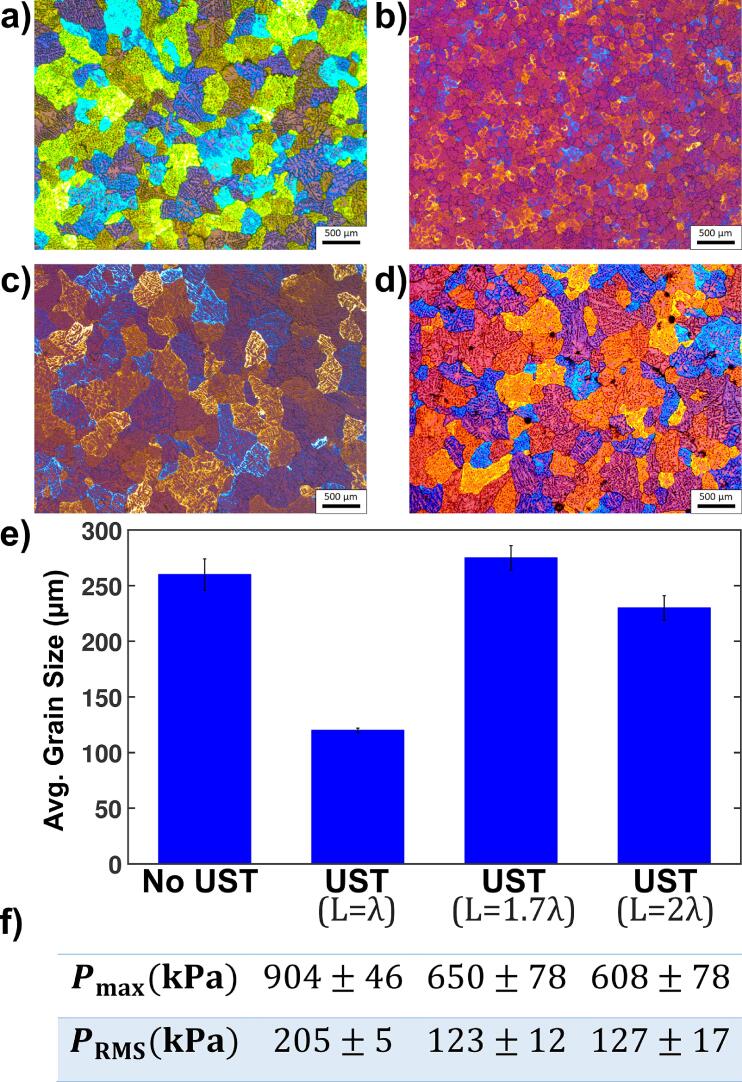
Typical anodized microstructures of samples in different tank lengths: (a) without UST, (b) with UST for L=λ, (c) with UST for L=1.7λ (off-resonance) and (d) with UST for L=2λ. (e) Bar plot of the average grain size for different processing condition. (f) $P_{max}$ and $P_{RMS}$ for microstructure experiments in liquid Al for the three different tank lengths. Note that these experiments were performed under the sonotrode (position P1).

Fig. 6 presents the $P_{max}$ (a) and $P_{RMS}$ (b) of liquid Al and DIW for different positions of the probe and all lengths of the vessel. For liquid Al, we observed a dependence of pressure on L, in particular for L>1λ, while the pressure was independent of the cavitometer position for L<1λ. This is seen for both $P_{max}$ and $P_{RMS}$. For DIW, however, we observed an opposite behaviour, as the pressure did not seem to depend on L at all, but indeed decreased as the cavitometer moved away from the cavitation zone, therefore, we observed the largest pressure magnitudes for DIW right under the sonotrode for any L value. This is, apparently, because the speed of sound in water in the cavitating environment changes significantly and makes the resonance conditions (λ) irrelevant (unlike in liquid aluminium as verified by microstructure analysis, see Section 3.3 and Fig. 7). Furthermore, the pressure magnitudes for liquid Al were much higher than those of water for any position or resonance length, except for L=2λ for which the pressures in liquid Al and water became comparable.

The magnitude of the acoustic pressures in a sonicated liquid also influences the cavitation activity. As seen in Fig. 6, the maximum pressure in liquid Al is more than 400 kPa for all positions and dimensions (except for L=2λ) within the vessel. Whereas, in case of DIW, the maximum pressure lies in the range of 100–200 kPa under and side of the ultrasonic source. This further supports the observation of Fig. 4, Fig. 5, where high frequency peaks close to 300 kHz were readily captured in case of liquid Al indicating the sustained oscillatory motion of cavitation bubbles. However, in the case of DIW, these high frequency peaks were supressed and only substantial peaks up to 100 kHz were captured, confirming that smaller cavitation bubbles (Table A2) undergo inertial oscillation and catastrophically collapse after a few acoustic cycles. Yasui et al. [45] reported that the energy of acoustic waves radiated from oscillating bubbles corresponding to resonance frequency peak at each acoustic cycle increases with the rise in pressure amplitude. The radiated acoustic energy further increases in bubble clouds within the liquid.

### 3.3 Microstructure analysis

Fig. 7 shows the microstructures (a–d) and their quantification in terms of average grain size (e), along with their corresponding acoustic pressure measurements under the sonotrode. It should be noted that for this investigation only the resonance lengths of λ and 2λ were considered (as in section 3.1) with the off-resonance length at 1.7λ for comparison. We chose L=1.7λ as an off-resonance comparison based on the previous work which shows a trend that larger partition distances increase the residence time [13]. In this work, we try to find an off-resonance length which still gives reasonable acoustic pressure, while obtaining longer residence time (Fig. 7(f)). Furthermore, L=1.7λ provides almost the same resonance ratio (0.85) to 2λ (which gives the maximum volume melt) as the ratio of L=λ (typically a desirable vessel length) to the off-resonance 1.1λ used in our acoustic pressure measurements in section 3.2.

The case of L<λ that showed the highest pressure in some locations (and discussed in Section 3.1 from a spectral point of view) has less practical meaning in a continuous casting environment as it significantly restricts the treated volume and especially reduces residence time as in [28] and thus it was not considered for microstructural analysis and comparison. The best result in terms of grain refinement (the indicator of UST efficiency) was obtained when UST was performed in the tank with L=λ (with grain size of 120 µm vs 260 µm for the untreated melt). This is consistent with the results of acoustic pressure measurements (Fig. 5, Fig. 6): tank with L=λ produced highest acoustic pressure (the maximum pressure for L=λ is 39% higher than that of L=1.7λ and 49% higher than L=2λ), because the tank length was at the main resonance mode where the cavitation activity was amplified [46] and the reflection from the tank walls was still quite strong (despite a larger bubble volume fraction as previously explained) as compared to when the tank walls were further apart. The important areas to consider for a homogeneous and effective treatment of the melt are the ones farther away from the obvious cavitation zone under the sonotrode, where cavitation treatment occurs anyway, and it seems that for the particular resonance wavelength L=λ the middle and edge points maintain the highest pressure magnitude, implying a more intense treatment throughout the melt volume. Furthermore, another advantage of L=λ, in contrast to the closely followed in pressure magnitudes L=0.5λ (Fig. 6), is that this particular configuration can treat 2 times more volume with the same pressure efficiency over the same period of time.

UST for L=2λ gave slight grain refinement (230 µm) reflecting the significant drop in the acoustic pressure (see Fig. 5, Fig. 6). Most interestingly, the lowest treatment efficiency was obtained when the tank is at off-resonance yet shorter length (L=1.7λ) producing average grain size of 275 µm which means no grain refinement at all (the average grain size is even larger than without UST, but can be considered the same within the statistical error). This hints at the presence of higher-order resonance at L=2λ which was observed in our previous work [12] and also here (Fig. 5). This also supports our assumption that the resonance conditions in liquid Al are more sustainable than in DIW, being less susceptible to the changes in acoustic properties induced by cavitation.

## 4 Conclusions

In this work, we measured experimentally the acoustic pressure induced by ultrasonic processing in both liquid Al and DI water with a broad frequency-calibrated cavitometer (8–400 kHz) for different tank lengths – in both resonant and non-resonant conditions – keeping all other dimensions constant and for various positions of the sensor. By conducting a comprehensive analysis based on a deconvolution algorithm, we reported detailed observations and comparison of the pressure spectra for various conditions. The effect of the ultrasonic treatment on liquid Al was verified by metallographic examination. The following conclusions can be drawn from this study:

1. The acoustic pressure in liquid Al can reach up to 600 kPa for relatively small lengths of the tank (L<2λ).
2. For liquid Al, the pressure field is sensitive to the length of the vessel and drops significantly for L=2λ (from 500 kPa to 200 kPa). Nevertheless, resonance conditions can be maintained in the liquid Al even in the presence of cavitation.
3. Significant high-frequency peaks (at around 300 kHz) are observed for liquid Al due to intensified pressure fields assumed to be caused by a large number of oscillating bubbles.
4. The acoustic pressure in DIW does not appear to depend on λ (presumably the speed of sound changes significantly upon cavitation and the resonance conditions are not met), but it depends on the position of the cavitometer for every given length, i.e. the pressure decays as the probe moves away from the sonotrode.
5. Microstructure observation through an averaged grain size analysis shows that the main mode of resonance (L=λ) gives the finest grain which also reflects the highest cavitation activity and treatment efficiency as compared to other conditions.

## CRediT authorship contribution statement

**Mohammad Khavari:** Conceptualization, Methodology, Formal analysis, Writing - original draft, Visualization, Software, Validation, Data curation. **:** . **Abhinav Priyadarshi:** Methodology, Resources, Investigation. **Tungky Subroto:** Methodology, Resources, Investigation. **Christopher Beckwith:** Investigation. **Koulis Pericleous:** Conceptualization, Writing - review & editing, Supervision, Funding acquisition. **Dmitry G. Eskin:** Conceptualization, Methodology, Resources, Writing - review & editing, Supervision, Funding acquisition. **Iakovos Tzanakis:** Conceptualization, Methodology, Resources, Writing - review & editing, Supervision, Funding acquisition.

## Declaration of Competing Interest

The authors declare that they have no known competing financial interests or personal relationships that could have appeared to influence the work reported in this paper.

## Figures and Tables

**Table A1:** Bubble radius for all peak frequencies in pressure spectrum for liquid Al, all tank lengths and three different probe positions.

L=0.5λ						L=λ					
Edge		Middle		Under		Edge		Middle		Under	
$f_{0}$ (kHz)	$R_{0}$ (μm)	$f_{0}$ (kHz)	$R_{0}$ (μm)	$f_{0}$ (kHz)	$R_{0}$ (μm)	$f_{0}$ (kHz)	$R_{0}$ (μm)	$f_{0}$ (kHz)	$R_{0}$ (μm)	$f_{0}$ (kHz)	$R_{0}$ (μm)
19.5	161.3	19.5	172.1	19.5	175.3	19.5	170.3	19.5	171.9	19.5	158.7
61.5	53.0	52.5	65.5	39	88.9	61	56.2	28	120.5	33	95.0
83.5	39.7	61.5	56.2	52.5	66.6	102.5	34.4	61	56.6	61	52.7
103	32.7	83.5	42.0	61.5	57.2	170	21.5	103	34.5	84	39.0
258.5	14.3	102	34.8	83.5	42.7	213	17.6	213	17.7	124	27.2
287.5	13.0	258.5	14.9	124	29.5	258.5	14.8	258.5	14.9	135	25.2
301.5	12.5	297	13.2	135	27.2	287.5	13.5	287.5	13.6	198	17.9
342.5	11.2	342.5	11.7	198	19.2	301.5	12.9	302	13.0	297.5	12.5
				297.5	13.3	327	12.1	327	12.1	330	11.5
				330	12.2	342.5	11.6	342.5	11.7		
						390.5	10.4	390.5	10.4		
L=1.1λ						L=2λ					

Edge		Middle		Under		Edge		Middle		Under	

$f_{0}$ (kHz)	$R_{0}$ (μm)	$f_{0}$ (kHz)	$R_{0}$ (μm)	$f_{0}$ (kHz)	$R_{0}$ (μm)	$f_{0}$ (kHz)	$R_{0}$ (μm)	$f_{0}$ (kHz)	$R_{0}$ (μm)	$f_{0}$ (kHz)	$R_{0}$ (μm)

19.5	161.4	19.5	164.8	19.5	170.4	19.5	134.9	19.5	135.5	19.5	138.7
65.5	50.0	28	115.6	33	101.8	58.5	47.5	58.5	47.7	39	71.2
88	37.8	39	83.8	39	86.5	98	29.7	98	29.8	68	42.3
103	32.7	48	68.6	63	54.5	213	15.1	169.5	18.4	124	24.6
169.5	20.7	65.5	50.9	84	41.4	258.5	12.9	213	15.2	135	22.8
200.5	17.9	79	42.6	117.5	30.3	287.5	11.8	258.5	12.9	156.5	20.0
213	16.9	93	36.6	124	28.8	301	11.3	287.5	11.8	198	16.4
258.5	14.3	103	33.3	135	26.6	326.5	10.6	300	11.4	297.5	11.6
287.5	13.0	117.5	29.4	156.5	23.2	342.5	10.2	342.5	10.2	330	10.7
301.5	12.5	160.5	22.1	188	19.7	390.5	9.2	390.5	9.2	379	9.5
327	11.7	169.5	21.1	198	18.8						
342	11.2	200.5	18.1	257.5	14.9						
379.5	10.3	213	17.2	297.5	13.1						
390.5	10.0	226	16.3	330	12.0						
		258.5	14.5	345.5	11.5						
		287.5	13.2	379	10.6						
		301	12.7								
		326.5	11.8								
		342.5	11.4								
		379.5	10.4								
		390.5	10.2								

**Table A2:** Bubble radii for all peak frequencies in pressure spectrum for DIW, all tank lengths and three different probe positions.

L=0.7λ						L=λ					
Edge		Middle		Under		Edge		Middle		Under	
$f_{0}$ (kHz)	$R_{0}$ (μm)	$f_{0}$ (kHz)	$R_{0}$ (μm)	$f_{0}$ (kHz)	$R_{0}$ (μm)	$f_{0}$ (kHz)	$R_{0}$ (μm)	$f_{0}$ (kHz)	$R_{0}$ (μm)	$f_{0}$ (kHz)	$R_{0}$ (μm)
19.5	133.1	19.5	131.0	19.5	138.9	19.5	128.4	19.5	129.2	19.5	135.6
58.5	46.9	58.5	46.3	26	105.1	58.5	45.6	54.5	48.9	48.5	56.8
102.5	28.2	102.5	27.9	39	71.3	102.5	27.5	102.5	27.6	70	40.4
213	15.0	213	14.9	48.5	58.0	118	24.3	118	24.4	84	34.2
259.5	12.7	259.5	12.6	70	41.2	213	14.7	213	14.8	108	27.3
287	11.7	287	11.6	84	34.9	259.5	12.5	259.5	12.5	124	24.2
299.5	11.3	299.5	11.2	108	27.8	287	11.5	287	11.5	135	22.4
328.5	10.5	328.5	10.4	124	24.6	299.5	11.1	299.5	11.2	198	16.1
342.5	10.1	342.5	10.1	135	22.8	342	10.0	342	10.0	210	15.3
				198	16.4					297.5	11.5
				297.5	11.6					330	10.6
				330	10.7						
L=1.3λ						L=2λ					

Edge		Middle		Under		Edge		Middle		Under	

$f_{0}$ (kHz)	$R_{0}$ (μm)	$f_{0}$ (kHz)	$R_{0}$ (μm)	$f_{0}$ (kHz)	$R_{0}$ (μm)	$f_{0}$ (kHz)	$R_{0}$ (μm)	$f_{0}$ (kHz)	$R_{0}$ (μm)	$f_{0}$ (kHz)	$R_{0}$ (μm)

19.5	127.0	19.5	128.6	19.5	137.8	19.5	126.5	19.5	128.3	19.5	135.7
30	84.1	30	85.1	26	104.4	30	83.8	30	85.0	26	102.8
48.5	53.6	54.5	48.7	48.5	57.6	54.5	48.0	54.5	48.6	48.5	56.9
58.5	45.1	102	27.7	70	41.0	58.5	45.0	66	40.8	70	40.5
102.5	27.3	118	24.3	84	34.7	70	38.2	102.5	27.5	84	34.3
213	14.6	200	15.5	124	24.5	97.5	28.4	118	24.3	108	27.3
287	11.5	213	14.7	135	22.7	213	14.6	213	14.7	124	24.2
299.5	11.1	259.5	12.5	198	16.3	299.5	11.0	259.5	12.5	135	22.5
342	9.9	287	11.5	297.5	11.6	342.5	9.9	287	11.5	198	16.1
		299.5	11.1	330	10.6			299.5	11.1	211.5	15.3
		342	10.0					342	10.0	297.5	11.5
										330	10.6

## Appendix

Fig. A1 shows the pressure distribution in the time domain (the final output of the deconvolution analysis) for a single random waveform for liquid Al for all cavitometer positions: edge (Fig. A1(a)), quarter length (Fig. A1(b)) and under the sonotrode (Fig. A1 (c)) each for three tank lengths L=0.5λ, λ and 2λ. The significant drop in the pressure magnitudes for 2λ is observed for all probe positions.

In Fig. A2, we compare the pressure in frequency domain for DIW and liquid Al for three cavitometer positions and different resonance lengths: off resonance (L=0.5λ for liquid Al and L=0.7λ for DIW, first row), L=λ (second row) and L=2λ (third row). For the smaller tanks, the pressure magnitudes in liquid Al were higher than those of DIW, as expected, but the difference increased as we moved away from the cavitation zone. However, the pressure magnitudes for DIW for all three positions were comparable with liquid Al for 2λ.

Table A1, Table A2 show the calculated bubble radii ($R_{0}$) from the Minnaert equation for the experimental conditions used for liquid Al and DIW, respectively.
